# Why Young Adults Obtain a Medical Marijuana Card: Associations with Health Symptoms and Heaviness of Use

**DOI:** 10.26828/cannabis/2021.01.001

**Published:** 2021-04-22

**Authors:** Justin F. Hummer, Rachana Seelam, Eric R. Pedersen, Joan S. Tucker, David J. Klein, Elizabeth J. D’Amico

**Affiliations:** 1 RAND Corporation, 1776 Main Street, PO Box 2138, Santa Monica, CA 90407; 2 University of Southern California, Keck School of Medicine, Department of Psychiatry and Behavioral Sciences, 2250 Alcazar Street, Suite 2200, Los Angeles, CA 90033

**Keywords:** medical marijuana, cannabis, legalization, physical health, mental health, sleep

## Abstract

**Objective.:**

Prior studies documenting more frequent and problematic use among young adults who have acquired medical marijuana (MM) cards have broadly compared those who use medically to those who use recreationally. Gaining a better picture of how health symptoms and problematic use vary both within those who have a MM card for specific condition domains and between those who do not have a MM card can provide key information for medical practitioners and states interested in adopting or updating MM policies.

**Method.:**

The current study categorizes young adults authorized to use MM into three mutually exclusive groups based on endorsements of qualifying conditions: (1) Physical Health only (e.g., AIDS, arthritis, cancer; *n* = 34); (2) Behavioral Health only (e.g., anxiety, depression, sleep problems; *n* = 75); and (3) Multiple Conditions (a physical and behavioral health condition; *n* = 71). Multiple and logistic regression models examined differences across marijuana use, problems, mental health, physical health, and sleep quality for MM condition categories and for those that only use marijuana recreationally (*n* = 1,015).

**Results.:**

After adjusting for socio-demographic factors (age, sex, sexual orientation, educational status, employment status, race/ethnicity, mother’s education, prior intervention involvement in youth), MM card holders, particularly those with physical health or multiple health conditions, reported heavier, more frequent, and more problematic and risky marijuana use compared to those using recreationally. Despite this pattern, those in different MM condition categories were generally not found to be more symptomatic in domains of mental or physical health relevant to their respective conditions, compared to different category groups or to those using recreationally.

**Conclusions.:**

Findings emphasize the importance of providers conducting a careful assessment of reasons for needing a card, along with use, to reduce potential harms while adding credibility to a medical movement with genuine promise of relief for many medical conditions.

The marijuana policy landscape continues to evolve in the direction of legalization, as more states grant access to marijuana for medical purposes (ProCon.org, 2020). As of May 2020, 33 states and the District of Columbia have legalized marijuana for medical purposes, and 11 states and D.C. have legalized it for recreational purposes. In this changing climate, marijuana use among young adults is of heightened scientific, clinical, and societal concern, particularly as more than half (54%) of young people in the U.S. initiate marijuana use by age 21 (Chen et al., 2017), and among those reporting past 30-day marijuana use, about one in five young adults (21–22%) meets diagnostic criteria for cannabis use disorder (CUD; Richter et al., 2016).

Given that most states have medical marijuana (MM) laws which allow purchase and use of marijuana, the number of people receiving a MM recommendation from their provider and enrolling in their state’s MM program has risen dramatically in the U.S. (Boehnke et al., 2019). Moreover, an increasing amount of research has been conducted into the medical effects of marijuana. In a comprehensive review, the National Academies of Sciences, Engineering and Medicine (NASEM) found conclusive or substantial evidence supporting that chronic pain, nausea and vomiting due to chemotherapy, multiple sclerosis spasticity symptoms, and short-term sleep outcomes among those with obstructive sleep apnea syndrome were improved as a result of marijuana treatment (NASEM, 2017). However, there was limited, insufficient, or no evidence of therapeutic value for many other conditions allowed under many state laws, including cancer, epilepsy, and irritable bowel syndrome. Research evidence on MM’s effects on mental health conditions, such as depression or anxiety, is also quite limited, and most of the broader indications for MM are supported by anecdote rather than controlled clinical trials (Wilkinson & D’Souza, 2014). Moreover, the limited data on clinical effectiveness should be weighed against the substantial evidence for marijuana-related harms, such as worsening respiratory symptoms, increased risk of motor vehicle accidents, lower birth rates, and increased risk for developing psychotic disorders (NASEM, 2017). More generally, marijuana’s long-term therapeutic benefits versus costs (including tolerance, addiction, and withdrawal) are not yet known (Brigden & England, 2018; Rhyne et al., 2016; Wilkinson et al., 2016).

Despite potential for medical benefits, there is growing evidence that young people enrolled in their state’s MM program (oftentimes designated by having a “MM card”) who report using marijuana for medical purposes are more likely to report heavy and problematic use than those who use marijuana recreationally (Choi et al., 2017; Tucker et al., 2019). Greater use may be expected among those who use marijuana medically because they presumably use on a regular schedule or have a medical regimen for use. It becomes more concerning when use becomes problematic. For example, cross-sectional data from the National Epidemiologic Survey on Alcohol and Related Conditions found higher rates of CUD among young adults reporting marijuana use for medical purposes compared to those who used it for nonmedical purposes (Choi et al., 2017). Crosssectional data from high school seniors in Monitoring the Future showed that those who obtained marijuana through their state’s MM programs also reported more frequent use and were more likely to report daily use and “being hooked” on marijuana compared with those obtaining marijuana from a nonmedical source (Boyd et al., 2015). Tucker and colleagues (2019) conducted a longitudinal study and found that young adults with a MM card were more likely to report heavier and more problematic use one year later, as well as a history of heavier use throughout adolescence compared to those without a MM card.

It is presumed that one obtains a MM card because of physical or mental health conditions. However, young people may obtain MM cards for access to marijuana, perhaps because they live in states that do not offer recreational marijuana or they are under 21 and could not purchase recreational marijuana even if it was available, or they can purchase marijuana more cheaply due to state taxes being lower for MM than recreational marijuana. These factors may motivate those who use more heavily to obtain a MM card. Indeed, one longitudinal study found that obtaining a MM card in young adulthood was primarily driven by frequency of use, rather than physical or mental health symptoms ostensibly associated with card acquisition (Pedersen et al., 2019).

Given these findings, research is needed to better understand young adults’ reasons for getting a MM card, the extent to which reasons coincide with reported mental and physical health symptoms, and whether young adults with a MM card report more mental and physical health symptoms than those who use marijuana without a MM card. Despite not being explicitly included in states’ list of qualifying conditions for MM, mental health conditions such as anxiety and depression are common reasons for using MM (Kosiba et al., 2019; Lankenau et al., 2018; Walsh et al., 2013, 2017), and multiple states allow MM use for these conditions through an additional “other symptoms” category that states consider as debilitating or having capacity to cause serious harm to an individual if not alleviated. In a study of young adults in Canada, self-reported mental health problems were higher among those who used MM, and MM card holders were more likely to report using marijuana to manage or improve mental health than those who did not use for medical reasons (Wadsworth et al., 2020). An interview-based study in California reported similar findings in a heavier using sample of young adults; those with a MM card were more likely to report a lifetime history of mental and physical health problems and to report using marijuana to relieve symptoms compared to those without a MM card (Lankenau et al., 2018). In contrast, a screening of general adult primary care patients who reported using marijuana found few distinct differences in medical, psychiatric, and non-marijuana substance use characteristics between those who used for medical compared to recreational purposes (Roy- Byrne et al., 2015). Although many people utilize MM to treat physical and mental health conditions, there is relatively little research evidence to date on the effects of marijuana on anxiety, depression and PTSD, and findings are much less robust than for physical conditions, notably chronic pain, epilepsy, and MS symptoms (Hill, 2015; NASEM, 2017; Stockings et al., 2018; Whiting et al., 2015). Given that the evidence of marijuana’s therapeutic benefit for mental health is not yet well-understood nor well-established, and that many young adults report using MM to manage mental health symptoms (Wadsworth et al., 2020), research is needed to understand 1) how mental health symptoms manifest for young people who use marijuana for different medical conditions, and 2) how this compares to those who use for nonmedical reasons.

## The Present Study

Prior studies are limited by broadly comparing those who use marijuana medically to those who use recreationally (e.g., Boyd et al., 2015; Choi et al., 2017; Lankenau et al., 2018; Roy-Byrne et al., 2015; Tucker et al., 2019), lack of random sampling (Lankenau et al., 2018), and lack of validated measures to assess symptom functioning (Lankenau et al., 2018; Wadsworth et al., 2020). Gaining a better picture of how health symptoms and problematic use vary both within those who have a medical card for *specific condition domains* and between those who do not have a MM card, using validated measures of functioning, can provide key information for medical practitioners and states interested in adopting or updating MM policies. The current study adds to this literature by categorizing young adults in California authorized to use MM into three mutually exclusive groups based on endorsements of (1) physical health conditions only (e.g., AIDS, arthritis, cancer), (2) behavioral health conditions only (e.g., anxiety, depression, sleep problems) or (3) multiple conditions (e.g., both a physical health and a behavioral health condition). We examined how these MM groups compared to each other, and how they compared to a large and racially/ethnically diverse sample of young adults who reported recreational marijuana use on their frequency and quantity of marijuana use, marijuana-related problems and risk, and several domains of functioning corresponding to conditions for which MM card holders acquired their cards (i.e., mental health, sleep quality, and physical health).

The premise that medical symptoms drive acquisition of a MM card should be reflected in endorsements of symptoms pertaining to card holders’ respective conditions, relative not only to those who use recreationally, but also relative to card holders with different conditions. Therefore, compared to other medical condition groups and to non-card holders, we expected that the Physical Health only group would report greater symptoms of poor physical health, whereas the Behavioral Health only group would endorse more mental health symptoms and worse sleep quality. In addition, those reporting multiple conditions for MM card acquisition were expected to be the most symptomatic in all health domains and to demonstrate the highest rates of use and problems. Overall, MM card holders across all health condition groups were expected to use marijuana more frequently, display more problematic and risky use, and be more symptomatic than non-card holders across all domains of functioning.

## METHODS

### Participants and Procedures

Participants are from a multiwave study of substance use. After being initially recruited in 6^th^/7^th^ grade for a substance use prevention program (CHOICE) conducted in 16 middle schools in southern California in 2008 (D’Amico et al., 2012), participants completed up to eleven annual surveys, with the first five middle school surveys conducted during physical education class and the rest of the surveys completed online. Further details of recruitment and retention rates across waves are described in detail elsewhere (Dunbar et al., 2018; D’Amico et al., 2016; D’Amico, Rodriguez et al., 2018). Briefly, at wave 6, when most participants transitioned out of middle school to over 200 high schools across the region, 61% of the sample was retained, and wave-to-wave retention rates from waves 7-11 ranged from 80-92%. Attrition from wave to wave was not associated with substance use. All study procedures were approved by the RAND institutional review board. Data for this study come from the online survey completed at wave 11 during 2018-2019 when participants were approximately 22 years old. Sale and possession of recreational marijuana became legal in California on November 8, 2016 and recreational marijuana outlets began opening on January 1, 2018. All data collection for the current study occurred after these legal milestones. Prior published studies utilizing data from this same cohort have examined associations between participant health characteristics, marijuana use, and MM card status (e.g., Pedersen et al., 2019). However, the interrelationships between these domains have not previously been evaluated using cohort data from the wave of the study presented herein.

### Measures

#### Socio-Demographics.

Participants self-reported age, sex at birth, sexual orientation (heterosexual/straight vs. gay/lesbian/bisexual/asexual/questioning), and race/ethnicity (“Which race/group best describes you? (Mark all that apply)”; mutually exclusive categories for Non-Hispanic White, Non-Hispanic Black, Hispanic, Non-Hispanic Asian, and Non-Hispanic Other/Multi-racial). We assessed mother’s education level (“How far did your mother go in school?”; didn’t finish high school, graduated from high school, some college, college degree or above) as a proxy for family socioeconomic status (Korupp et al., 2002). Participants reported current college enrollment (“Describe your current education setting”; currently in graduate school or college or technical/trade school vs. all other responses) and employment status (“Are you currently working at a paid job (including self-employment)?”; employed part-time or employed full-time vs. unemployed and looking for a job right now or unemployed and not looking for a job).

#### MM Card Status and Conditions.

Participants who reported use of marijuana on at least one day in the past year were asked whether they currently had a MM card (yes/no). If they selected yes, they were then asked: “For what condition(s) have you been provided with a medical marijuana card?” Response options included all qualifying conditions to become a medical marijuana patient in California according to Proposition 215, with revised Senate Bill (SB) 420. Conditions include: AIDS, anorexia, arthritis, cachexia, cancer, chronic pain, glaucoma, migraine, persistent muscle spasms, seizures, severe nausea. In addition, SB 420 includes a provision for “any other chronic or persistent medical symptom that either substantially limits a person’s ability to conduct one or more of major life activities as defined in the Americans with Disabilities Act of 1990, or if not alleviated, may cause serious harm to the person’s safety, physical, or mental health.” Given high rates of endorsement for mental health and sleep in the research literature, three additional items were included to capture the behavioral health domain: depression, anxiety, and sleep problems. Lastly, to account for the provision of the Senate Bill around any other chronic or persistent symptoms, participants could write in a condition. Card holders were asked to ‘select all that apply,’ and were categorized into mutually-exclusive groups based on endorsement of conditions: Physical Health (AIDS, anorexia, arthritis, cachexia, cancer, chronic pain, glaucoma, migraine, persistent muscle spasms, seizures, severe nausea); Behavioral Health (anxiety, depression, sleep problems). Open-ended responses were included into categories as follows: Physical Health (e.g., back pain; fibromyalgia; period pains; sciatica) and Behavioral Health (e.g., panic attacks). Those who endorsed conditions from both Behavioral Health and Physical Health were put into a Multiple Conditions category. Young adults who reported past year use but did not currently have a MM card were categorized into the Non-Medical group.

Two participants who reported being provided a MM card but did not endorse any of the health conditions were excluded from the analyses. In the resulting final sample of those reporting past year marijuana use (*n* = 1,195), 15.1% (*n* = 180) reported having a MM card. Most of the sample belonged to the non-medical group (84.9%; *n* = 1,015). Of those with a MM card, 41.7% (*n* = 75) endorsed only a behavioral health condition as the reason for which they were provided a MM card, 18.9% (*n* = 34) endorsed only physical health, and 39.4% (*n* = 71) endorsed both a physical health and a behavioral health condition as reasons for which they were provided a MM card.

#### Marijuana Use.

*Frequency of marijuana use* was assessed with a single item on number of days used marijuana in the past month (0-30 days). Participants also indicated how many times they use marijuana on the days they use it (Bogart et al., 2005; Ellickson et al., 2005). *Quantity of marijuana use* focused on flower/bud, asking, “On the days you use marijuana, on a typical use day, how much marijuana flower/bud do you personally consume?” (Kilmer et al., 2013). Response options ranged from 1 = “Less than 0.25g” to 10 = “More than 5g,” and were re-coded using the mid-point of each response option to represent quantities in grams (e.g., “between 1 and 1.5g” re-coded to 1.25g) with a final range from 0.25 to 5 grams. The majority (84.1%; %*n* = 1005) of the sample endorsed a quantity of flower/bud consumed on a typical use day; thus, we retained this information in our analyses. *Multiple episodes of use per day* on days marijuana was used was assessed with the question “On the days you use marijuana, how many times do you use it?”. Because the majority of responses were “once” per day, we dichotomized this item as once vs. more than once.

#### Marijuana-related problems.

The *Cannabis Use Disorders Short Form* (CUDIT-SF; Bonn- Miller et al., 2016) asks participants how often during the past 6 months they found they were not able to stop using marijuana/cannabis once they had started; devoted a great deal of their time to getting, using, or recovering from marijuana/cannabis; and had a problem with their memory or concentration after using marijuana/cannabis (rated 0 = never to 4 = daily or almost daily; α = 0.74). *Marijuana consequences* were assessed with ten items asking frequency of negative outcomes in the past year due to their marijuana use, rated from 1 = never to 7 = 20 or more times. (e.g., “you had less motivation to do things because of using marijuana”) (Bogart et al., 2005; Ellickson et al., 2005; Simons et al., 2012). Items were summed to create a composite score (α = 0.90). Separate items for *marijuana-related problem behaviors* asked how often in the past year participants had driven a car, motorcycle or other vehicle after using marijuana; had been a passenger in a car or other vehicle with a driver who had been drinking alcohol or using drugs; and sold marijuana or hashish (grass, pot, weed) (1 = not at all to 6 = 20 or more times). Because they are rare events, these three items were dichotomized into indicators for any occurrence.

#### Behavioral Health.

The Patient Health Questionnaire (PHQ-8; Kroenke et al., 2009) assessed eight depression symptoms (e.g., “feeling, down, depressed or hopeless”) in the past two weeks (α = 0.91). The Generalized Anxiety Disorder scale (GAD-7; Spitzer et al., 2006) assessed seven anxiety symptoms (e.g., “feeling nervous, anxious, or on edge”) experienced in the past two weeks (α = 0.94). Items in both scales were rated from 0 = not at all to 3 = nearly every day, and composite scores were created by summing items. Overall sleep quality in the past month was measured with a single item from the Pittsburg Sleep Index (Buysse et al., 1989) on a scale from 1 = very bad to 4 = very good.

#### Physical Health.

A composite score for physical health was generated from three items: the single item of the General Health factor on the 12-item Short-Form Health Survey (Ware et al., 1996) assessing “In general, would you say your health is…” with response options ranging from 1 = excellent to 5 = poor, and two items from the PROMIS Pediatric Physical Function Scales (DeWitt et al., 2011) (e.g., “In the past month…I have been physically able to do the activities I enjoy most”) with response options of 1 = with no trouble to 5 = not able to do. Items were reverse scored with higher scores reflecting better physical health (α = 0.79).

### Analytic Plan

Several variables followed non-normal distributions in which more than half of responses contained the same value. These variables were more appropriate for logistic regression and dichotomized prior to analysis. The remaining outcomes approximated normal distributions and were deemed suitable for the robust nature of linear regression. Multivariable linear or logistic regressions with follow-up post-hoc tests with Tukey adjustment for multiple comparisons were conducted to compare the four mutually exclusive groups (MM card for physical health condition only; MM card for behavioral health only; MM card for multiple conditions; and no MM card) on frequency and quantity of marijuana use, marijuana-related problems, physical health, mental health, and sleep measures. Group comparisons controlled for socio-demographic covariates: age, sex (male vs. female), sexual orientation (straight vs. other), college status (in college vs. not), employment status (currently employed vs. not), race/ethnicity (Non-Hispanic White, Non-Hispanic Black, Non-Hispanic Asian, Non-Hispanic other race, Hispanic), mother’s education (less than high school, high school, some college, college or above), and CHOICE intervention status.

## RESULTS

The analytic sample for this study (Table 1) was comprised of young adults who reported past year marijuana use (*n* = 1,195). They were 21.6 years old on average (SD = 0.8), 46% male (*n* = 547); 80.8% reported being heterosexual (*n* = 966), 61.0% reported being in college at the time of survey administration (*n* = 730), 73.1% reported being employed (*n* = 872), and 52.4% of participants reported that their mothers had completed college (*n* = 627). The sample was racially and ethnically diverse; 42% reported being Hispanic (*n* = 503), 26.8% non-Hispanic White (*n* = 321), 2.6% non-Hispanic Black (*n* = 31), 16.0% non-Hispanic Asian (*n* = 191), and 12.6% reported being some other race or multi-racial (*n* = 151).

### Marijuana use.

A full description of regression and post-hoc tests for group differences can be found in Table 2, with statistically significant findings summarized here. Those with no MM card reported significantly less frequent marijuana use in the past month relative to those in the Behavioral Health Only group (6.2 days vs 15.3 days, Tukey-adjusted *p* < 0.001), the Physical Health Only group (6.2 days vs. 12.0 days, *p* = .005), and the Multiple Conditions group (6.2 days vs. 18.4 days, *p* < .001). Furthermore, those in the Multiple Conditions group reported more frequent use compared to the Physical Health Only group (18.4 days vs. 12.0 days, *p* = .01). For using multiple times on days used, we found similar patterns: those with no MM card were less likely to use marijuana multiple times per day than those in the Behavioral Health Only group (39% vs 77%, *p* = <.001), the Physical Health Only group (39% vs. 64%, *p* = .04), and the Multiple Conditions group (39% vs. 79%, *p* < .001). A similar pattern was also seen for quantity of marijuana flower/bud consumed on a typical use day, with those in the no MM card group reporting consuming less flower/bud than the Physical Health Only group (0.7g vs. 1.3g, *p* < .001), the Behavioral Health Only group (0.7g vs. 1.1g, *p* < .001), and the Multiple Conditions group (0.7g vs. 1.3g, *p* < .001).

### Marijuana-related problems.

We also found differences between the no MM card group and the other condition groups for past year marijuana consequences and the CUDIT-SF score. Those with no MM card reported fewer marijuana-related consequences in the past year than those in the Physical Health Only group (15.5 vs. 21.7, *p* < .001) and those with Multiple Conditions (15.5 vs. 18.5, *p* = .03). Those with no MM card also reported lower CUDIT-SF scores than those in the Behavioral Health Only group (1.5 vs. 2.5, *p* = .006) and the Multiple Conditions group (1.5 vs. 2.7, *p* = .01).

For marijuana-related problem behavior outcomes, we found some differences. Those with no MM card reported fewer instances of ever driving under the influence of marijuana than those in the Physical Health Only group (24% vs. 53%, *p* = .004) and the Multiple Conditions group (24% vs. 49%, *p* <.001). The Behavioral Health Only group was also less likely to report any driving under the influence of marijuana (25%) than the Physical Health Only (*p* = .04) or Multiple Conditions group (*p* = .02). The same pattern was found for selling any marijuana in the past year: those with no MM card reported fewer instances of selling marijuana than those in the Physical Health Only group (7% vs. 32%, *p* < .001) and the Multiple Conditions group (7% vs. 29%, *p* < .001). Additionally, the Behavioral Health Only group was also less likely to report selling any marijuana in the past year (10%) than Physical Health Only (*p* = .03) or Multiple Conditions (*p* = .02). Finally, we found that those with no MM card were more likely to report ever having been a passenger in a car or other vehicle with a driver who had been drinking alcohol or using drugs than the Behavioral Health Only group (43% vs. 27%, *p* = .03); the Behavioral Health Only group also reported fewer such incidents than Physical Health Only (27% vs. 54%, *p* = .04).

### Mental and physical health and sleep.

Only one significant group difference was found for mental health: those with no MM card reported lower scores on the PHQ-8 than those in the Multiple Conditions group (6.1 vs. 7.8, *p* = .048). There were no significant differences between groups on physical health. Finally, those in the Physical Health Only group reported significantly better overall sleep quality relative to those in the No MM Card group (3.2 vs. 2.8, *p* = .05) as well as Multiple Conditions group (3.2 vs. 2.7, *p* = .01).

## DISCUSSION

The current study provides an in-depth look at differences across marijuana use, problems, mental and physical health, and sleep for MM card holders who have a card for different conditions, and for those who only use marijuana recreationally. As expected, young adult MM card holders reported heavier and more frequent marijuana use, including days of use, multiple episodes of usage on days used, and quantity on use days, than those who did not have a MM card. MM card holders reported using marijuana on at least twice as many days in the past month and used approximately twice the amount of marijuana flower/bud compared to non-card holders. Drilling down by condition type, those who used marijuana to manage multiple behavioral and physical health conditions reported the most days of use in the past month; however, the three condition groups did not differ in the likelihood of using multiple times per day. Young adult MM card holders, particularly those with physical health only or multiple conditions, also reported more problematic and risky use of marijuana compared to those using recreationally. Those in the physical health condition group and those with multiple conditions reported greater marijuana-related consequences compared to those without a MM card, and a greater likelihood of driving after using marijuana compared to the behavioral health condition group. Further, the physical health condition group and those with multiple conditions also reported selling marijuana more frequently than those in the non-card or behavioral health groups, replicating prior research (Tucker et al., 2019). Results of the CUDIT-SF revealed that the behavioral health condition group, the physical health condition group, and the multiple conditions group all had mean scores above 2.0, which has been found to reliably identify 78% of individuals who meet criteria for CUD according to DSM-5 (Bonn-Miller et al., 2015). When comparing group differences, only those with behavioral health or multiple conditions had a significantly higher severity score than those with no MM card. Thus, it appears that the most problematic use occurs among young adults who report physical health or multiple health conditions. Overall, findings highlight the importance for providers to probe why young adults may want to obtain a card given that card holders were generally more likely to meet the threshold of CUD. Thus, screening for both reasons for providing the card and current marijuana use may provide an opportunity for brief intervention if needed. This is particularly important as recent studies find that teens age 14-18 who report numerous marijuana consequences and/or who have a diagnosis of CUD responded positively to a 15-minute brief motivational intervention, reporting less cannabis use and consequences one year later (D’Amico, Parast, et al., 2018; D’Amico et al., 2019).

Interestingly, although MM card holders reported heavier and more problematic marijuana use than non-card holders, they were generally not found to be more symptomatic in the mental and physical health domains that were assessed. Specifically, compared to all other groups, the behavioral health group did not report greater symptoms of depression, anxiety, or sleep quality, and the physical health condition group did not report worse physical health. Those reporting multiple conditions did, however, report greater depressive symptoms than those without a card, and they also reported worse sleep quality than those with physical health conditions. Although there are physical health conditions that can benefit from marijuana use (NASEM, 2017), several studies show that in states with medical laws, many people who use medicinally also use marijuana recreationally (Lankenau et al., 2018; Pacula et al., 2016; Walsh et al., 2013). Moreover, a study of primary care patients who reported using marijuana found few distinct differences in medical, psychiatric, care utilization, and nonmarijuana substance use characteristics between those who used medically compared to those who did not (Roy-Byrne et al., 2015), and in a study of young adult MM patients, 15% admitted that their physician recommendations for the card were based on a fabricated health problem (Lankenau et al., 2018). Thus, some clinicians, media, and policymakers question whether people using marijuana for medical purposes are really different from those using marijuana for recreational purposes, which contributes to suspicion by some healthcare providers that MM is a way to increase the likelihood for legalizing recreational use (Pedersen & Sandberg, 2013) or, for individuals who use, a path to obtain marijuana more cheaply and in higher quantities.

The concerns noted above must be weighed with attendant consideration to research design. For example, the overall pattern of our findings supports prior research showing greater frequency of use among young people with access to MM (Boyd et al., 2015; Tucker et al., 2019), and adds to these findings by categorizing individuals according to their condition clusters and evaluating several domains of functioning. However, findings do not represent definitive evidence of a lack of heightened symptoms pertaining to the condition for which individuals acquire a MM card given that our selected measures captured broad aspects of symptoms across these domains. Further research is needed to understand how the condition for which an individual receives a MM card maps onto specific symptomatology. In addition, due to the cross-sectional design, it is not possible to determine the extent to which marijuana is adequately treating the symptoms associated with card acquisition. Even in the absence of a MM card, “self-medicating” with marijuana—using marijuana to ease physical or psychological symptoms without direction or authorization from a licensed physician—is common among young people (Bottorff et al, 2009). We did not assess motives for use among those without a card and many of those individuals may also be using marijuana for specific symptom relief; yet it is not without risks. Perhaps most notable is the evidence concerning psychosis. A recent review found that of 13 prospective longitudinal studies, 10 showed that those who use cannabis had a significantly increased risk of psychosis compared with those who do not, while 2 of the remaining 3 showed a trend in the same direction (Sideli et al., 2020). Marijuana use may also exacerbate other mental health symptoms. A meta-analysis on longitudinal studies of marijuana and depression found that heavy marijuana use may be associated with increased risk of depression (Lev-Ran et al., 2014). Furthermore, individuals who use marijuana may experience acute adverse effects, such as anxiety (Hall & Weier, 2015), which could be contrary to what they aim to achieve through use (Schofield et al., 2006; Walsh et al., 2013). Thus, for those not already doing so, providers who write prescriptions for MM should consider screening for common mental health problems, weighing the potential benefits with potential contraindications of recommending marijuana as a treatment, and make appropriate alternative referrals to mental health specialists as warranted.

It’s important to note our study limitations. First, although we measured several domains, our constructs of physical and mental health were not exhaustive. It is possible that individuals within condition groups may vary on other measures, such as pain interference or other dimensions of mental and physical health. We also acknowledge that the two-week time period of mental health symptom assessment is brief, despite this being the standardized time frame for these measures. Given this, we may not have had enough sensitivity within the time window to detect longer-term fluctuations of mental health symptoms. Third, sample sizes within condition groups were somewhat small and varied; however, proportions were expected given smaller numbers of those who use marijuana medically (6.2%) in the U.S. relative to those who use recreationally (90.2%) or those who use both medically and recreationally (3.6%) (Compton et al., 2017). Fourth, the cross-sectional nature of this study prohibits definitive conclusions regarding cause and effect, and longitudinal research is needed to assess whether symptoms reported by MM card holders may be improving with use. Finally, the study relied on subjective rather than objective reporting of symptoms and reasons for acquiring a MM card. More precise conclusions can be drawn through combined use of self-reported information along with medical documentation from treating physicians and/or medical records (see Nunberg et al., 2011).

Despite limitations, findings add to our understanding of young adults’ reasons for getting a MM card and highlight the ways in which those with varying conditions compare on frequency and quantity of use, problematic and risky use, and on mental and physical health symptoms. Perhaps most notably, we found that MM card holders did not report greater severity of mental and physical health symptoms than those without a card. Many individuals struggle with legitimate medical and psychological concerns that can benefit from MM (NASEM, 2017). If marijuana is to be used for such purposes, it should be subjected to the same evidence-based review and regulatory policies as those used for other pharmaceutical agents prescribed by physicians. Our current findings emphasize the importance of providers conducting a careful assessment of the reasons for needing a card, along with use, given that those with a card tend to use more frequently and heavily and report more problems. This could help reduce potential harms due to heavy use and contraindications (such as use at a young age among those at risk for schizophrenia and other forms of mental illness), while also adding credibility to a medical movement with genuine promise of relief for many medical conditions.

## Figures and Tables

**Table 1.** Sample Descriptives for Young Adults Reporting Past Year Marijuana Use

Age, *mean (SD)*	21.6 (0.8)
Male gender, *n* *(%)*	547 (45.7%)
Sexual orientation, *n* *(%)*	
Heterosexual/straight	966 (80.8%)
Gay/lesbian/bisexual/asexual/questioning	229 (19.2%)
In college, *n* *(%)*	730 (61.0%)
Employed, *n* *(%)*	872 (73.1%)
Race/ethnicity, *n* *(%)*	
Non-Hispanic White	321 (26.8%)
Non-Hispanic Black	31 (2.6%)
Hispanic	503 (42.0%)
Non-Hispanic Asian	191 (16.0%)
Non-Hispanic Other/Multi-racial	151 (12.6%)
Mother’s education, *n* *(%)*	
Did not finish high school	158 (13.2%)
High school	177 (14.8%)
Some college	165 (13.8%)
College	627 (52.4%)
Don’t know	70 (5.9%)
CHOICE intervention, *n* *(%)*	632 (52.8%)
Medical marijuana card, *n* *(%)*	180 (15.1%)
Conditions endorsed for being provided with a medical marijuana card, *n* *(%)*	
Physical health condition only	34 (2.9%)
Behavioral health condition only	75 (6.3%)
Multiple health conditions (at least one physical health and one behavioral health condition)	71 (5.9%)
**Outcomes**	
Frequency of marijuana use in past month, *mean (SD)* [range 0-30]	7.7 (10.5)
Number of times using marijuana on days used, *mean (SD)* [range 0-63]	2.5 (4.0)
Cannabis Use Disorders Identification Test Short Form score, *mean (SD)* [range 0-12]	1.7 (2.6)
Marijuana consequences in past year, *mean (SD)* [range 2-70]	16.0 (9.4)
Number of times in past year having driven a car, motorcycle, or other vehicle after using	2.2 (5.3)
marijuana, *mean (SD)* [range 0-20]	
Number of times in past year having been a passenger in a car or other vehicle with a driver who	2.3 (4.6)
has been drinking alcohol or using drugs, *mean (SD)* [range 0-20]	
Generalized Anxiety Disorders scale-7, *mean (SD)* [range 0-21]	5.6 (5.4)
Patient Health Questionnaire-8, *mean (SD)* [range 0-24]	6.2 (5.6)
Physical health composite, *mean (SD)* [range 0-12]	9.1 (2.3)
Sleep quality, *mean (SD)* [range 1-4]	2.8 (0.8)

**Table 2.** Between Group Differences on Study Outcomes

	Health Condition Categories													
Linear Regression	Physical Health (PH)			Behavioral Health (BH)			Multiple Conditions (MC)			Non Card-holders (NC)			F test (3df)	Significant Differences Between Groups
	Mean	LCL	UCL	Mean	LCL	UCL	Mean	LCL	UCL	Mean	LCL	UCL		

Past Month MJ Use	12.0	8.7	15.4	15.3	13.0	17.5	18.4	16.1	20.6	6.2	5.6	6.8	51.76***	NC < PH, BH, MC BH < MC
Quantity of MJ Flower/Bud	1.3	1.0	1.6	1.1	0.9	1.3	1.3	1.1	1.5	0.7	0.6	0.7	21.47***	NC < PH, BH, MC
MJ Consequences	21.7	18.5	24.8	17.0	15.0	19.1	18.5	16.4	20.7	15.5	14.9	16.0	7.28***	NC < MC, PH
CUDIT-SF	2.4	1.6	3.3	2.5	1.9	3.1	2.4	1.8	3.0	1.5	1.3	1.7	7.03***	NC < BH, MC
PHQ-8 Severity	6.9	5.0	8.8	6.9	5.6	8.1	7.8	6.5	9.1	6.1	5.7	6.4	2.75*	NC < MC
GAD-7 Severity	4.7	2.9	6.5	5.9	4.7	7.1	6.9	5.7	8.1	5.5	5.1	5.8	2.01	
Physical Health Composite	8.8	8.0	9.6	8.7	8.2	9.3	8.7	8.2	9.3	9.1	9.0	9.3	1.37	
Sleep Quality	3.2	2.9	3.4	2.8	2.7	3.0	2.7	2.5	2.8	2.8	2.8	2.8	3.14*	NC, MC < PH
**Logistic Regression**	%	LCL	UCL	%	LCL	UCL	%	LCL	UCL	%	LCL	UCL	X^2^ test (3df)	

Use MJ Multiple Times/Day	64%	46%	79%	77%	66%	85%	79%	68%	87%	39%	36%	42%	67.29***	NC < PH, BH, MC
Riding With Impaired Driver	54%	36%	70%	27%	18%	38%	43%	32%	54%	43%	40%	46%	9.12*	BH < PH, NC
Drove After Using MJ	53%	35%	70%	25%	17%	36%	49%	37%	60%	24%	21%	27%	28.40***	NC, BH < MC, PH
Sold MJ/Hashish	32%	18%	50%	10%	5%	18%	29%	20%	41%	7%	6%	9%	48.09***	NC, BH < MC, PH

* *p* < 0.05,

** *p* < 0.01,

*** *p* < 0.001

*Note.* These tests to compare group differences are adjusted for by: age, sex, sexual orientation, college status, employment status, race/ethnicity, mother’s education, and CHOICE intervention status. LCL = lower control limit; UCL = upper control limit; MJ = marijuana; CUDIT-SF = cannabis use disorders identification test short form; PHQ = patient health questionnaire; GAD = generalized anxiety disorders.
